# Oxygen isotope anomaly in tropospheric CO_2_ and implications for CO_2_ residence time in the atmosphere and gross primary productivity

**DOI:** 10.1038/s41598-017-12774-w

**Published:** 2017-10-13

**Authors:** Mao-Chang Liang, Sasadhar Mahata, Amzad H. Laskar, Mark H. Thiemens, Sally Newman

**Affiliations:** 1 Research Center for Environmental Changes, Academia Sinica, Taipei, Taiwan; 2 Graduate Institute of Astronomy, National Central University, Taoyuan, Taiwan; 3 Department of Chemistry and Biochemistry, University of California at San Diego, La Jolla, USA; 4 Division of Geological and Planetary Sciences, California Institute of Technology, Pasadena, USA

## Abstract

The abundance variations of near surface atmospheric CO_2_ isotopologues (primarily ^16^O^12^C^16^O, ^16^O^13^C^16^O, ^17^O^12^C^16^O, and ^18^O^12^C^16^O) represent an integrated signal from anthropogenic/biogeochemical processes, including fossil fuel burning, biospheric photosynthesis and respiration, hydrospheric isotope exchange with water, and stratospheric photochemistry. Oxygen isotopes, in particular, are affected by the carbon and water cycles. Being a useful tracer that directly probes governing processes in CO_2_ biogeochemical cycles, Δ^17^O (=ln(1 + δ^17^O) − 0.516 × ln(1 + δ^18^O)) provides an alternative constraint on the strengths of the associated cycles involving CO_2_. Here, we analyze Δ^17^O data from four places (Taipei, Taiwan; South China Sea; La Jolla, United States; Jerusalem, Israel) in the northern hemisphere (with a total of 455 measurements) and find a rather narrow range (0.326 ± 0.005‰). A conservative estimate places a lower limit of 345 ± 70 PgC year^−1^ on the cycling flux between the terrestrial biosphere and atmosphere and infers a residence time of CO_2_ of 1.9 ± 0.3 years (upper limit) in the atmosphere. A Monte Carlo simulation that takes various plant uptake scenarios into account yields a terrestrial gross primary productivity of 120 ± 30 PgC year^−1^ and soil invasion of 110 ± 30 PgC year^−1^, providing a quantitative assessment utilizing the oxygen isotope anomaly for quantifying CO_2_ cycling.

## Introduction

The net growth rate and level of CO_2_ in the atmosphere represents a dynamic balance between anthropogenic activities and natural sources and sinks^1^. The diurnal and seasonal cycles, however, are largely affected by terrestrial photosynthesis and respiration^2–4^. The oxygen isotopic composition of atmospheric CO_2_ has been shown to be a powerful tracer for improving understanding of carbon and water cycles involving CO_2_ (ref.^2–9^), providing a unique way to estimate terrestrial gross primary productivity^2,5,8,9^. Oxygen has three stable isotopes (^16^O,^17^O, and^18^O). The^18^O/^16^O isotope ratio is used widely for studying aspects of the carbon and water cycles of natural systems^2,3,5–9^, but^17^O/^16^O has rarely been used owing to added analytical difficulty. Here, we compare and analyze the results of the triple-oxygen isotope composition of surface air CO_2_ from northern hemisphere sites in the western Pacific (South China Sea and Taipei, Taiwan)^10^ and available data from the middle east (Jerusalem, Israel)^11^ and western North America (La Jolla, United States)^12^, to provide deeper insight into the global CO_2_ cycle of the atmosphere, biosphere, and hydrosphere (The data from Göttingen, Germany^13^ are not included because of the presence of unknown drift in their two-year ^17^O data, though their first year of data agree well with the data reported in this work. Our approach is discussed in detail in a following section). We derive the residence time of CO_2_ in the atmosphere and gross primary production (GPP) from the integrated data set and discuss how interhemispheric transport affects these quantities. Given that the tropospheric mixing time in each hemisphere is much shorter than the interhemispheric mixing time^14,15^ and the latter shorter than the CO_2_ residence time derived here (see below), the compiled data should be a valid approximation of the global average.

We show that the terrestrial flux (the CO_2_ cycling flux between the atmosphere and terrestrial biosphere) can be quantified using the Δ^17^O values of CO_2_, where

1 $${{\rm{\Delta }}}^{17}{\rm{O}}=\,\mathrm{ln}(1+{{\rm{\delta }}}^{17}{\rm{O}})-{\rm{\lambda }}\times \,\mathrm{ln}(1+{{\rm{\delta }}}^{18}{\rm{O}}),$$

and we set λ = 0.516 (a value commonly used in the literature^16–18^) and δ’s are referenced with respect to a commonly used scale, V-SMOW. Here, we follow typical notation (equation 1) to report the values of Δ^17^O. However, for the budget calculation that involves multiple-component mixing, Δ^17^O is not a conserved quantity^19^, and instead, the linear form of Δ^17^O, *Δ*, is used:

2 $${\Delta }={{\rm{\delta }}}^{17}{\rm{O}}-{\rm{\lambda }}\times {{\rm{\delta }}}^{18}{\rm{O}}.$$

The budget formulation is then identical to that using δ’s. We note that for describing sources in the budget calculation, Δ^17^O and *Δ* are equally valid. For example, global meteoric water^20^ obeys the relation λ = 0.528, and the reported Δ^17^O value for δ^18^O greater than −10‰ is 0.032 ± 0.017‰. In contrast, *Δ* = 0.026 ± 0.017‰. The means are different but the standard deviations are the same, demonstrating that over the range of the δ^18^O values considered, there is no advantage of utilization of either equation (1) or (2) for source representation.

The advantage of using Δ^17^O (or*Δ*) over δ^18^O measurements is that Δ^17^O directly probes the associated processes in the carbon and water cycles^11,20–25^, as discussed in the next section. Moreover, λ, unlike δ, is insensitive to temperature, and both δ^17^O and δ^18^O are affected following the canonical mass-dependent relation^23,26^. Exchanging oxygen isotopes with water is the major process that we consider in determining CO_2_ fluxes between the atmosphere and biosphere/hydrosphere; the associated λ is well defined experimentally^11,23^, and the fluxes (e.g., the terrestrial flux - the cycling flux between the terrestrial biosphere and atmosphere, inferred from the oxygen isotopic composition of CO_2_) are robustly constrained (cf. ref.^8^).

A classic application of the triple isotope approach is the measurement of Δ^17^O in dissolved O_2_ in waters^25,27,28^. The biologically produced Δ^17^O value of O_2_ is balanced by the anomaly produced in the middle atmosphere as a consequence of O_2_-O_3_-CO_2_ photochemistry. Since the signal has a millennium time scale, it can be used to study biospheric changes during the past thousand years. In addition, there is a potentially analogous application for CO_2_, provided processes that affect Δ^17^O in CO_2_ are quantified. Similar to O_2_, the atmospheric Δ^17^O of CO_2_ is controlled by CO_2_-O_2_-O_3_ photochemistry and various anthropogenic, biospheric, and hydrospheric processes, including fossil fuel burning, photosynthesis, respiration, and exchange with leaf and soil water, oceans and other bodies of water^11,16,18,19,29–33^. The primary enhancing source of the oxygen isotopic anomaly resides in the middle atmosphere, as a consequence of the exchange reaction between CO_2_ and O_3_ via the excited state oxygen atom O(^1^D) (ref.^29–32^). Along with various sources^19^ and processes^5,9,11,23,33^ that determine the “net” Δ^17^O of CO_2_ emitted from the Earth’s surface, Hoag and co-authors^16^ investigated the contribution of stratospheric CO_2_ to the tropospheric CO_2_ mass-independent isotopic composition and predicted an anomaly of Δ^17^O ≈ 0.15‰, above the mean value of emissions/fluxes from the surface.

## Sources and processes defining Δ^17^O values

The size of the anomaly is dependent upon the choice of λ and the reference scale. Here, we choose 0.516 for the slope and the most commonly used scale for oxygen, V-SMOW. We note that the selection of reference scale does not affect interpretation, provided the variation and partitioning among the three oxygen isotopes are properly accounted for. Given that the carbon flux estimation presented in this paper is based on the deviations of the oxygen anomalies of reservoirs/processes from that measured in atmospheric CO_2_, it is natural to take the λ value describing the variation of the triple-oxygen isotopic partitioning in tropospheric CO_2_. Processes that affect CO_2_ isotopologues in the troposphere are terrestrial, oceanic and anthropogenic, with the first being dominant. In the terrestrial biosphere, leaf water transpiration governs the variation of oxygen isotopes in CO_2_; the mean, however, is largely determined by water-CO_2_ equilibration catalyzed by carbonic anhydrase^2,3,5–7,9,33^. It has been found previously that the transpiration λ value is a function of air relative humidity^21^, whereas dependence on other meteorological variables such as temperature and soil water isotopic composition has not been observed. We set λ = 0.516, as it represents the transpiration λ at 75% relative humidity, a globally averaged humidity near the surface^34^. This λ value is essentially the same as that of CO_2_ we obtained (0.518 ± 0.004, see below) for the western Pacific, which had an average relative humidity of 76 ± 4% during 2010–2015 (data obtained from the Center for Weather Bureau, Taiwan; site code: 466920; the value changes slightly to 72 ± 11% if considering only day time between 6 AM to 6 PM). However, given the sparse spatiotemporal coverage of the data, the governing slope cannot be firmly decided. More data taken under a variety of environmental conditions are needed to set a better constraint. From the current understanding of the processes occurring, we consider plant transpiration the most important process affecting the variation of atmopsheric CO_2_ (e.g., see Landais *et al*.^21^ and Cuntz *et al*.^3^ and references contained therein). We stress that the value of λ does not affect the flux interpretation shown below, as long as equation (2) is used, but the selection must best represent the variation of atmospheric CO_2_.

**Figure 1:**
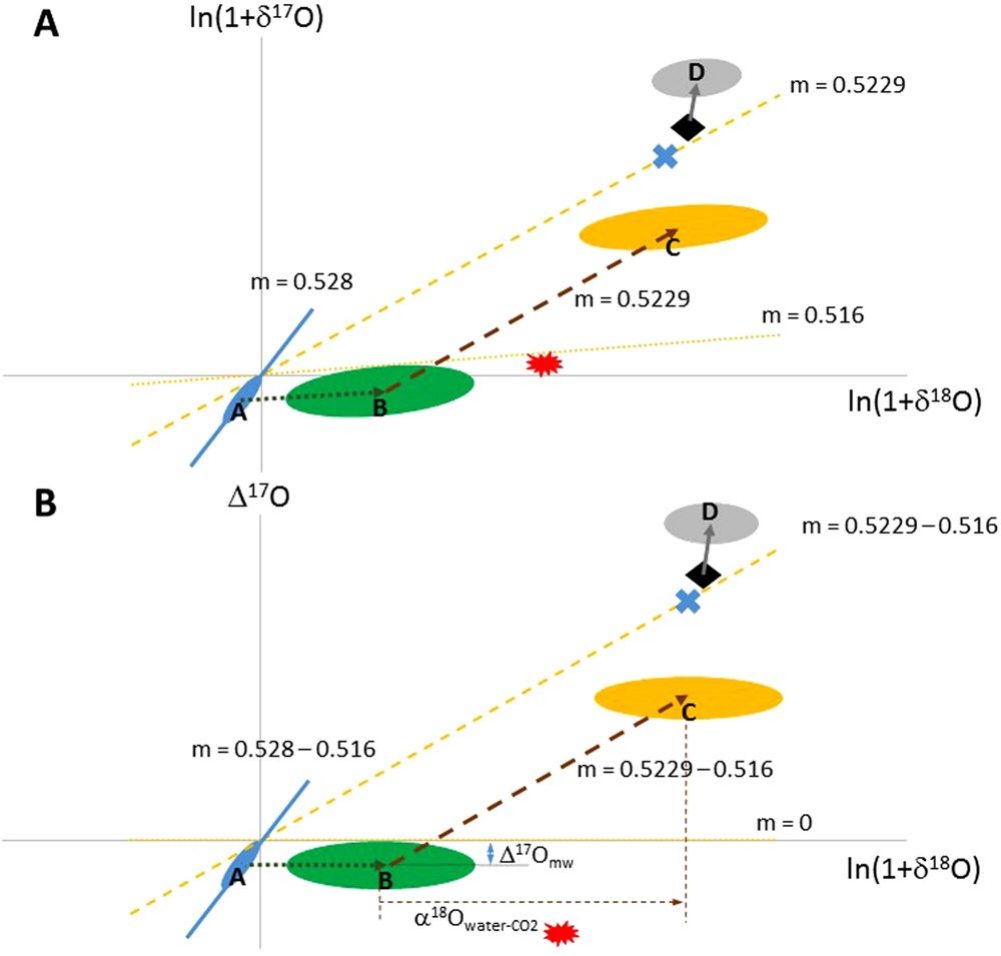
Schematic diagram (not to scale) of the sources and transport of CO_2_ considered in this paper. (**A**) ln(1 + δ^17^O) vs. ln(1 + δ^18^O) plot for meteoric water (blue), transpiration water (green), plant equilibrated CO_2_ (yellow), and stratospheric modified CO_2_ (grey). Ocean water equilibrated CO_2_ is shown by the blue “X” and averaged tropospheric CO_2_ value by the diamond. Anthropogenic CO_2_ is denoted by the explosive red starburst symbol. Arrows indicate transport. The slopes (m) for the lines AB, BC, and diamond-D are 0.516, 0.5229, and > 1, respectively. See text for details. (**B**) Similar to (**A**) but for Δ^17^O vs. ln(1 + δ^18^O). The corresponding slopes have been decreased by 0.516. Δ^17^O_mw_ is the meteoric Δ^17^O, the y-intercept of the line AB. α^18^O_water-CO2_ represents the fractionation in δ^18^O of water and CO_2_.

**Figure 2:**
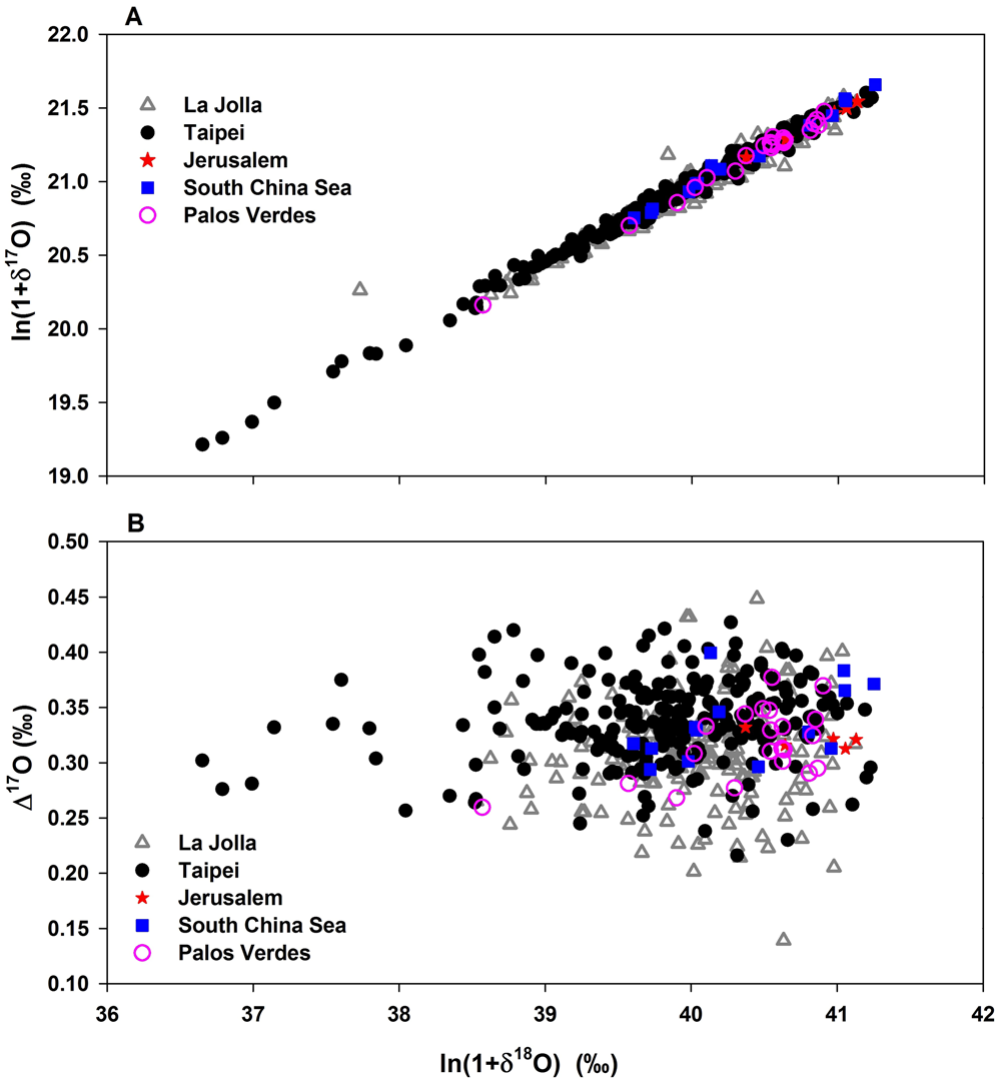
(**A**) ln(1 + δ^17^O) vs. ln(1 + δ^18^O) plot for atmospheric CO_2_ collected from Taipei (Taiwan), South China Sea, La Jolla (United States), and Jerusalem (Israel). Values in ‰ are referenced to V-SMOW. The geometric mean regression of the Taipei data gives ln(1 + δ^17^O) = (0.519 ± 0.005)× ln(1 + δ^18^O) + (0.2 ± 0.2‰). (**B**) The reported Δ^17^O values vs. ln(1 + δ^18^O). Note that the Δ^17^O values for the last two datasets have been re-scaled following equation (1). The error bars are smaller than the symbol size, with an error of ~0.05‰ for δ^18^O and ~0.01‰ for Δ^17^O. The two points (give values or reference to table where the data are given) from La Jolla beyond the plotting range of Δ^17^O are not shown.

A schematic diagram (not to scale) that describes various sources and processes modifying CO_2_ isotopologues is shown in Fig. 1, which summarizes the oxygen isotope transport at steady state. On a global scale, equilibrium processes are the major controllers in oxygen isotope dynamics; we show below in the Box model section that kinetic fractionations are insignificant. (Previous work^13^ utilized a time-dependent model showing a measurable seasonal cycle of Δ^17^O at an amplitude of ~0.05‰. The present work assesses the global carbon budget at steady state, leaving the assessment of spatiotemporal variability, including seasonality, to a latter paper when data covering a variety of spatial and temporal scales are available). Three sources/processes are considered: terrestrial (meteoric) water, ocean water, and anthropogenic CO_2_, with the last inheriting the atmospheric O_2_ isotopic composition. The variation of the triple oxygen isotopic composition of meteoric water follows a slope of 0.528 (ref.^20^). Subsequent isotopic exchange between water and atmospheric CO_2_ modifies the oxygen isotopic composition of CO_2_ following a slope of 0.5229 (ref.^11^). In addition, in the terrestrial biosphere, plant transpiration changes the source water following a slope of 0.516, a value chosen to represent the average slope at the globally averaged relative humidity of 75% (ref.^21,34^), as discussed above. These three values (0.528, 0.5229, and 0.516) determine the Δ^17^O value of CO_2_ mediated by processes involving water. We use the scheme in Fig. 1 to follow changes in oxygen isotopic composition, starting with meteoric water (point A). Transpiration that affects leaf water changes source meteoric water from A to B; the line AB follows the slope of 0.516. Subsequent water-CO_2_ isotopic exchange determines the composition of oxygen in leaf CO_2_, following the slope of 0.5229 to point C. For exchange with ocean water, only water-CO_2_ equilibration is involved, that changes CO_2_ to the blue “X.” When the CO_2_ enters the stratosphere, the coupled CO_2_-O_2_-O_3_ photochemistry moves the tropospheric CO_2_ to D; this path goes through mean tropospheric CO_2_ (diamond) and has slope of >1 (ref.^29–32^). Large-scale circulation and synoptic eddy mixing bring the modified CO_2_ back to the troposphere. The tropospheric CO_2_ (diamond) represents a balance among stratospheric (grey), terrestrial (yellow), oceanic (blue “X”), and the final component - anthropogenic CO_2_ (red symbol). Figure 1B, plotting Δ^17^O versus ln(1 + δ^18^O), shows that our reference λ is chosen (0.516) so that plant transpiration does not change the Δ^17^O value of terrestrial water. This is a rotation of Fig. 1A such that a slope of 0.516 becomes 0, and this removes the dominant variation along the correlation in Fig. 2A, as shown in Fig. 2B.

## Δ^17^O values of source CO_2_

In addition to well-quantified photochemical processes in the middle atmosphere, there are two known processes that modify Δ^17^O: combustion and isotope exchange with water. The former produces CO_2_ with Δ^17^O = −0.21‰, the same as air O_2_ (ref.^19^), used for the anthropogenic component below.

**Figure 3:**
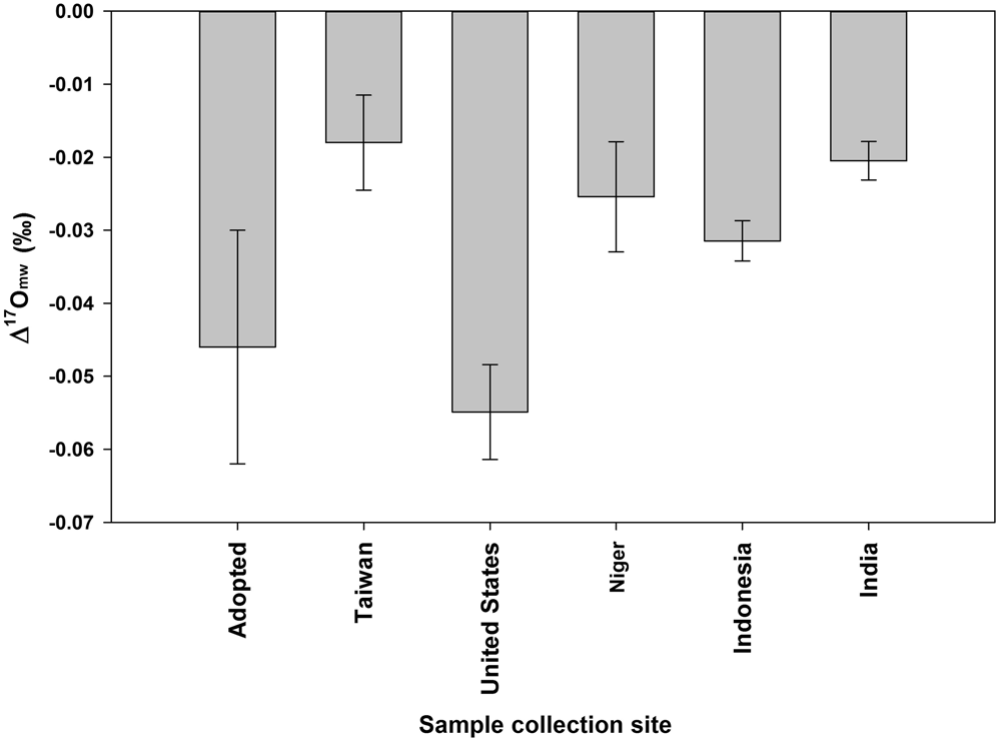
The Δ^17^O value (Δ^17^O_mw_) of global meteoric water^20^ adopted in this work. For comparison, the values in Taiwan^28^, mainland USA^48^, and three tropical countries (Niger^49^, Indonesia^20^, and India^20^) are also shown.

Isotopic exchange with water can be estimated using water-CO_2_ equilibrium^11,23^. Under the assumed definition of λ derived from equation (1), Δ^17^O of CO_2_ emitted from sources involving water-CO_2_ equilibration processes like respiration and soil invasion, following the slope of 0.5229 (ref.^11^), is (0.5229 − 0.516) × ln $({{\alpha }^{18}}_{{\rm{w}}{\rm{a}}{\rm{t}}{\rm{e}}{\rm{r}}-{{\rm{C}}{\rm{O}}}_{2}})\,+\,{{\rm{\Delta }}}^{17}{O}_{{\rm{m}}{\rm{w}}}$ (see the previous section and Fig. 1), where ${{\alpha }^{18}}_{{\rm{w}}{\rm{a}}{\rm{t}}{\rm{e}}{\rm{r}}-{{\rm{C}}{\rm{O}}}_{2}}$ and Δ^17^O_mw_ are, respectively, the fractionation factor for water equilibrated ^18^O in CO_2_ and Δ^17^O of water. See Fig. 1B for the schematics. We adopt ${{\alpha }^{18}}_{{\rm{w}}{\rm{a}}{\rm{t}}{\rm{e}}{\rm{r}}-{{\rm{C}}{\rm{O}}}_{2}}\,=\,1.043$ at a globally averaged land temperature of 15 °C (taken over 60° south to 75° north, where most biological activities occur; ref.^34^). Globally averaged meteoric water has Δ^17^O_mw_ = −0.046 ± 0.005‰ (1 standard error; or 0.032 ± 0.003‰ at λ = 0.528; ref.^20^), excluding highly depleted waters having δ^18^O less than −10‰ in high latitude regions covered by snow and/or ice. (Here, standard error represents the error of a sample mean; standard deviation describes the error of a single measurement, the spread of replicate analyses of a single sample, or the spread of an ensemble). Figure 3 shows that the values of Δ^17^O_mw_ from various regions are comparable.

The Δ^17^O value for terrestrial CO_2_ (Δ^17^O_land_) is calculated to be 0.244 ± 0.005‰ ((0.5229 − 0.516) × ln(1.043) − 0.046‰ = 0.000244 = 0.244‰). We note that since water transpiration in plants follows λ = 0.516 at 75% relative humidity^21^, this process does not change Δ^17^O values of waters originating from meteoric water. This equivalence is a major advantage of choosing λ = 0.516 in equation (1). (We note that 5% variation in relative humidity results in ~0.015‰ change in the Δ^17^O of leaf water-equilibrated CO_2_, through water transpiration^21^).

The other largest water reservoir is the oceans. The globally averaged Δ^17^O of ocean waters (Δ^17^O_ow_) is 0.000 ± 0.001‰ (or −0.005 ± 0.001‰ at λ = 0.528; ref.^20^), and the resulting Δ^17^O of oceanic CO_2_ at 20 °C (ref.^35^) is 0.284 ± 0.001‰. The observation that Δ^17^O of meteoric water is lower than that of oceanic water suggests that the Δ^17^O approach has greater sensitivity to terrestrial processes than to oceanic ones.

Below we combine the values calculated above with data from several locations around the world (La Jolla, CA, USA; Jerusalem, Israel; Taipei, Taiwan; and the South China Sea) to put constraints on GPP and the oxygen isotope residence time of CO_2_ in the atmosphere. The oxygen isotope residence time is defined by the ratio of the atmospheric CO_2_ mass loading (*M*) and the CO_2_ mass flux between the atmosphere and biosphere/hydrosphere (F_sur_). The flux is inferred from the mass balance calculation obtained using the triple oxygen isotopic composition of tropospheric CO_2_ shown below.

## Methods

In addition to using data available in the literature from Jerusalem^11^, La Jolla^12^, and western Pacific regions^10^, we have continuously collected air for isotopic analysis of CO_2_ in three locations: (1) Academia Sinica campus (abbreviated AS; 121°36′51″ E, 25°02′27″ N; ~10 m above ground level or 60 m above sea level) in Taipei, Taiwan and (2) the campus of National Taiwan University (NTU; 121°32′21″ E, 25°00′53″ N; ~10 m above ground level or 20 m above sea level; ~10 km southwest of Academia Sinica). To check the reported Δ^17^O values in the eastern Pacific^12^, we have also collected and analyzed CO_2_ from Los Angeles, California at a latitude slightly higher than La Jolla, along the coast on Palos Verdes peninsula (118°10.9′ W, 33° 44.7′ N; PVD). Data reported in this work and analyzed in Taiwan are provided in full in the supplementary material.

Analytical methods are described in detail elsewhere^10,18,36–38^ and summarized here. Air from western Pacific regions for isotope analysis was collected in pre-conditioned 1-liter Pyrex bottles, achieved by passing dry, high purity nitrogen through the bottles overnight. The sampling bottles used for concentration (~350-ml bottle) and isotope (1-liter) analyses were connected in series. Samples were collected and compressed to 2-bar after flushing the bottles for 5 minutes with ambient air at a flow rate of ~2 liter per min. Moisture was removed during sampling using magnesium perchlorate to minimize subsequent isotope exchange between CO_2_ and water^39^. Concentration of CO_2_ is measured using a LI-COR infrared gas analyzer (model 840A, LI-COR, USA), with reproducibility better than 1 ppmv. The PVD samples were collected on Saturday afternoons at about 14:00 PST, into 2-liter evacuated Pyrex flasks after passing through Mg(ClO_4_)_2_. Carbon dioxide was separated from the air samples cryogenically and measured, following the method described in Newman *et al*.^38^. The CO_2_-O_2_ oxygen isotope exchange method developed previously^36,37^ was used to measure the Δ^17^O of CO_2_ samples. Isotopic analyses were done using a FINNIGAN MAT 253 mass spectrometer in the dual inlet mode. The analytical precision obtained for a single measurement of the Δ^17^O value of CO_2_ is better than 0.01‰ (1-σ standard deviation).

## Results

The concentrations of the isotopologues of CO_2_ at South China Sea (SCS) are close to those reported at Mauna Loa measured by the National Oceanic and Atmospheric Administration - Earth System Research Laboratory (data available online at http://www.esrl.noaa.gov/gmd/dv/iadv/), with [CO_2_] 395.4 ± 7.3 ppmv and δ^13^C −8.47 ± 0.22‰ (1-σ standard deviation). In Taipei (AS + NTU), the averaged [CO_2_] is 416.2 ± 18.3 ppmv, δ^13^C is −9.22 ± 0.83‰, and δ^18^O is 40.65 ± 0.82‰. [CO_2_] varies between ~350 and 475 ppmv, with low values during the day and high at night, representing the combined effect of natural biogeochemical cycle (photosynthesis and respiration), anthropogenic emissions, and boundary mixed layer height diurnal variation^38^. During day time hours, photosynthesis dominates, resulting in reduction of CO_2_ concentration and less negative δ^13^C and δ^18^O values. The CO_2_ content is lower during the day than night also due to dilution as the boundary layer deepens. The mean values obtained for [CO_2_] and δ^13^C, as compared to SCS, show that in Taipei a clear contribution from anthropogenic emissions is seen. Given the proximity of the AS and NTU stations (~10 km apart), air transport time is shorter than the CO_2_ oxygen isotope residence time shown below, resulting in, on a yearly basis, similar levels of CO_2_ isotopologues, including Δ^17^O.

Figure 2A compiles tropospheric CO_2_ data in a plot of ln(1 + δ^17^O) vs. ln(1 + δ^18^O). The least square linear regression analysis of the data obtained in Taipei yields a slope of 0.518 ± 0.004 (excluding some outliers at δ^18^O < 38.5‰) and intercept of 0.3 ± 0.2‰ (1 standard error, R^2^ = 0.99). The observation that the same slope is obtained as for transpiration at ~75% relative humidity (close to the value at the sampling sites in western Pacific, 76 ± 4% averaged between 2010 and 2015) suggests that transpiration is likely a controlling process affecting the variation of the triple oxygen isotopic composition of near surface CO_2_. As the variation of oxygen isotopic compositions in CO_2_ at the two Taipei sampling sites is biogeochemically mediated, one may use Δ^17^O to estimate the actual “flux” between the atmosphere and soil/leaf, which in turn gives the value for GPP. The overall ratio of ln(1 + δ^17^O)/ln(1 + δ^18^O) for Taipei is 0.524 ± 0.001, consistent with that of a water-CO_2_ equilibrium value of 0.5229 ± 0.0001 (ref.^11,23^), further verifying that the oxygen of near-surface CO_2_ in this region is primarily affected by biogeochemistry with minor influences from the stratosphere and human activities. The Δ^17^O values in Taipei vary from 0.216 to 0.415‰ (Fig. 2B) with an average of 0.335 ± 0.039‰ (1-σ standard deviation of the range), a value similar to 0.31 ± 0.06‰ obtained in La Jolla^12^ and 0.321 ± 0.007‰ in Jerusalem^11^. (Note that the values for the last two have been re-scaled with respect to λ = 0.516, for the sake of consistency among the data sets). The value at SCS is 0.335 ± 0.033‰. The value reported at La Jolla is the lowest among the four. We then check the possibility that the difference between La Jolla and Taiwan is caused by the difference in Δ^17^O scale between the two labs. By taking CO_2_ samples collected also in the eastern Pacific region but at a higher latitude near a southwest facing beach on the Palos Verdes peninsula (PVD). The obtained values for 2015 are 0.317 ± 0.032‰, essentially the same as the decadal mean from La Jolla. The average of the mean Δ^17^O values from La Jolla, Jerusalem, Taipei, and the South China Sea (0.326 ± 0.005‰) is used below in the box model calculation of GPP and oxygen isotope residence time. The averaged Δ^17^O value decreases by only 0.004‰ if just the two largest datasets, from Taiwan and La Jolla, are averaged. Therefore, we note that whether the SCS, Jerusalem, and PVD data are included does not change the conclusion presented below. For example, a 0.01‰ reduction in Δ^17^O results in ~0.2-year decrease in the oxygen isotope residence time. Moreover, given that the oxygen isotope residence time is on the order of one year only and sources and processes that change the isotopologues of the tropospheric CO_2_ are variable spatially and temporally, spatiotemporal inhomogeneity in Δ^17^O is expected. The average of the mean values from more locations should be more representative for the global Δ^17^O.

## Box model

A box model is employed to assess various contributing processes for Δ^17^O. At steady state, the mass balance equation for δ (where δ is either δ^17^O or δ^18^O), following Cuntz *et al*.^3^, can be written as follows:

3 $$\begin{array}{c}{C}_{{\rm{a}}}{M}_{{\rm{a}}}\frac{{{\rm{d}}\delta }_{{\rm{a}}}}{{\rm{d}}{\rm{t}}}=0={\varepsilon }_{{\rm{l}}}({{\rm{F}}}_{{\rm{l}}{\rm{a}}}-{{\rm{F}}}_{{\rm{a}}{\rm{l}}})+{{\rm{F}}}_{{\rm{l}}{\rm{a}}}({\delta }_{{\rm{l}}}-{\delta }_{{\rm{a}}})+{{\rm{F}}}_{{\rm{r}}}\,({\delta }_{{\rm{r}}}-{\delta }_{{\rm{a}}}+{\varepsilon }_{{\rm{s}}})+{{\rm{F}}}_{{\rm{s}}}\,({\delta }_{{\rm{s}}}-{\delta }_{{\rm{a}}})\\ \quad \quad \quad \quad \quad +{{\rm{F}}}_{{\rm{a}}{\rm{o}}}({\delta }_{{\rm{o}}}-{\delta }_{{\rm{a}}}+{\varepsilon }_{{\rm{o}}})+{{\rm{F}}}_{{\rm{a}}{\rm{n}}{\rm{t}}{\rm{h}}}({\delta }_{{\rm{a}}{\rm{n}}{\rm{t}}{\rm{h}}}-{\delta }_{{\rm{a}}})+{{\rm{F}}}_{{\rm{s}}{\rm{t}}}({\delta }_{{\rm{s}}{\rm{t}}}-{\delta }_{{\rm{a}}})\end{array}$$

where *C* is the volume mixing ratio of CO_2_, *M* is the mass of the atmosphere, subscripts “a”, “st”, “anth”, “l”, “r”, “s”, and “o” of δ’s represent the δ values of the sampled air, the stratosphere, anthropogenic emissions, and leaf, respiration, soil, and ocean water, respectively, ε’s are the associated kinetic fractionation factors, and F is the flux in and out of a reservoir such that the subscript “la” refers to leaf-to-air, “al” air-to-leaf, and “ao” air-to-ocean. We then rewrite the equation (3) in terms of *Δ* in steady state as follows.

4 $$\begin{array}{c}{{\rm{F}}}_{{\rm{l}}{\rm{a}}}({{\rm{\Delta }}}_{{\rm{l}}}-{{\rm{\Delta }}}_{{\rm{a}}})+{{\rm{F}}}_{{\rm{r}}}\,({{\rm{\Delta }}}_{{\rm{r}}}-{{\rm{\Delta }}}_{{\rm{a}}})+{{\rm{F}}}_{{\rm{s}}}\,({{\rm{\Delta }}}_{{\rm{s}}}-{{\rm{\Delta }}}_{{\rm{a}}})+{{\rm{F}}}_{{\rm{a}}{\rm{o}}}({{\rm{\Delta }}}_{{\rm{o}}}-{{\rm{\Delta }}}_{{\rm{a}}})\\ \quad \quad \quad \quad \quad +{{\rm{F}}}_{{\rm{a}}{\rm{n}}{\rm{t}}{\rm{h}}}({{\rm{\Delta }}}_{{\rm{a}}{\rm{n}}{\rm{t}}{\rm{h}}}-{{\rm{\Delta }}}_{{\rm{a}}})+{{\rm{F}}}_{{\rm{s}}{\rm{t}}}({{\rm{\Delta }}}_{{\rm{s}}{\rm{t}}}-{{\rm{\Delta }}}_{{\rm{a}}})=\,0,\end{array}$$

where the kinetic terms ((F_la_ − F_al_) × ε_l_ × (λ_l_ − λ_0_) + F_r_ × ε_s_ × (λ_s_ − λ_0_) + F_ao_ × ε_o_ × (λ_o_ − λ_0_)) become negligible (even with extreme values for F at 500 PgC year^−1^ or λ_l,s,o_ at 0.529, the value for equilibrium water between condensed and vapor phases, the isoflux is found to be less than 1‰ PgC year^−1^). We note that *Δ* in the linear definition in equation (4) obeys mass conservation whereas Δ^17^O in the logarithmic definition does not. The use of Δ^17^O in equation (4) results in an error about 10% in each term derived, i.e., ~40 PgC year^−1^ biased too high in F_la_ + F_r_ + F_s_ and ~0.2 year too short in the resident time of CO_2_ in the atmosphere (though still within the error of the estimation). Parameters and values used in the box modeling are summarized in Table 1.

**Table 1:** Summary of the parameters and values considered in the box modeling. The quoted error refers to 1 standard error, representing the error of a sample mean.

Parameter	Description	Value chosen	Notes
RH	near surface air relative humidity	75 ± 5%	Estimated; Dai^34^
${{\alpha }^{18}}_{{{\rm{w}}{\rm{a}}{\rm{t}}{\rm{e}}{\rm{r}}-{\rm{C}}{\rm{O}}}_{2}}$	equilibrium fractionation	1.043	Brenninkmeijer *et al*.^35^; at 15 °C
${{\alpha }^{18}}_{{{\rm{w}}{\rm{a}}{\rm{t}}{\rm{e}}{\rm{r}}-{\rm{C}}{\rm{O}}}_{2}}$	equilibrium fractionation	1.042	Brenninkmeijer *et al*.^35^; at 20 °C
λ_0_	nominal λ	0.516	Adopted
${\lambda }_{{{\rm{w}}{\rm{a}}{\rm{t}}{\rm{e}}{\rm{r}}-{\rm{C}}{\rm{O}}}_{2}}$	water-CO_2_ equilibrium λ	0.5229	Barkan and Luz^11^
λ_trans_	transpiration λ	0.516 ± 0.004	Taken at RH = 75 ± 5% relative humidity; Landais *et al*.^21^ and Dai^34^
λ_diff_	diffusion λ	0.5185	Barkan and Luz^22^
λ_l_	cross-leaf λ	~0.5–0.53	Unknown
λ_s_	respiration λ	~0.5–0.53	Unknown
λ_o_	cross-ocean λ	~0.5–0.53	Unknown
Δ^17^O	atmospheric CO_2_ Δ^17^O	0.326 ± 0.005‰	n = 4; measured from four locations
Δ^17^O_mw_	meteoric water Δ^17^O	−0.046 ± 0.005‰	n = 40; Luz and Barkan^20^
Δ^17^O_ow_	oceanic water Δ^17^O	0.000 ± 0.001‰	n = 38; Luz and Barkan^20^
Δ^17^O_anth_	anthropogenic CO_2_ Δ^17^O	−0.21‰	Laskar *et al*.^19^
Δ^17^O_l_	leaf water CO_2_ Δ^17^O	0.244 ± 0.005‰	Calculated
Δ^17^O_r_	respiration CO_2_ Δ^17^O	0.244 ± 0.005‰	Calculated
Δ^17^O_s_	soil CO_2_ Δ^17^O	0.244 ± 0.005‰	Calculated
Δ^17^O_land_	terrestrial CO_2_ Δ^17^O	0.244 ± 0.005‰	Calculated
Δ^17^O_o_	oceanic CO_2_ Δ^17^O	0.284 ± 0.001‰	Calculated
ε_l_	kinetic fractionation, ε, in ^18^O for CO_2_ diffusion in and out of stomata	−7.4‰	Farquhar *et al*.^9^
ε_s_	ε for CO_2_ diffusion out of soil	−7.2‰	Miller *et al*.^50^
ε_o_	ε for CO_2_ diffusion in and out of ocean surface	0.8‰	Vogel *et al*.^51^
F_la_	leaf-to-air flux	to be determined	
F_al_	air-to-leaf flux	to be determined	
F_s_	soil invasion	to be determined	
F_r_	respired flux	F_al_ − F_la_	
F_oa_	ocean-to-air flux	90 ± 6 PgC year^−1^	n = 3; IPCC^1,3,40^
F_ao_	air-to-ocean flux	90 ± 6 PgC year^−1^	n = 3; IPCC^1,3,40^
F_land_	terrestrial flux	F_al_ + F_s_ = 345 ± 70 PgC/ year^−1^	Derived in this work
F_sur_	surface flux	F_land_ + F_oa_	
F_anth_	anthropogenic flux	9.4 ± 0.8 PgC year^−1^	for year 2011; IPCC^1^, Peters *et al*.^41^
F_st_ × (Δ^17^_st_ − Δ^17^_a_)	stratospheric isoflux	50 ± 3‰ PgC year^−1^	n = 5; Liang *et al*.^17^
NEP	net ecosystem productivity	10 PgC year^−1^	Saugier *et al*.^46^
*κ*_*c*_	stomatal conductance	1.33–2.97	Cuntz *et al*.^3^; Farquhar *et al*.^9^
*θ*_*eq*_	degree of hydration	0.7–0.78	Farquhar *et al*.^9^; Gillon and Yakir^33^; Cousins *et al*.^44^
GPP	gross primary productivity	(F_al_ − F_la_)/0.88	Ciais *et al*.^2^

Finally, we have *Δ*_l_ = −0.009 ± 0.006‰, *Δ*_r_ = *Δ*_s_ = 0.019 ± 0.006‰, *Δ*_o_ = 0.075 ± 0.001‰, and *Δ*_anth_ = −0.286 ± 0.001‰ (or Δ^17^O_l_ = Δ^17^O_r_ = Δ^17^O_s_ = Δ^17^O_land_ = 0.244 ± 0.005‰, Δ^17^O_o_ = 0.284 ± 0.001‰, and Δ^17^O_anth_ = −0.213 ± 0.001‰). The oceanic flux F_oa_ is 90 ± 6 PgC year^−1^ (an average of IPCC 2001, 2007, and 2013; ref.^1–3,40^), F_anth_ is 9.4 ± 0.8 PgC year^−1^ for year 2011 (ref.^1,41^). We further take F_la_ ≈ 2F_r_ ≈ 2F_s_ (from the fact that global net productivity is much less than the gross productivity, and the assumption of catalyzed soil invasion^7^), and with this, the terrestrial flux F_land_ equal to F_la_ + F_r_ + F_s_. Then equation (4) can be reduced to

5 $${{\rm{F}}}_{{\rm{land}}}\times ({{\Delta }}_{{\rm{l}}}\,-\,{{\Delta }}_{{\rm{a}}}+{{\Delta }}_{{\rm{s}}}\,-\,{{\Delta }}_{{\rm{a}}})/2+{{\rm{F}}}_{{\rm{ao}}}\times ({{\Delta }}_{{\rm{o}}}\,-\,{{\Delta }}_{{\rm{a}}})+{{\rm{F}}}_{{\rm{anth}}}\times ({{\Delta }}_{{\rm{anth}}}\,-\,{{\Delta }}_{{\rm{a}}})+{{\rm{F}}}_{{\rm{st}}}\times ({{\Delta }}_{{\rm{st}}}\,-\,{{\Delta }}_{{\rm{a}}})=0.$$

**Figure 4:**
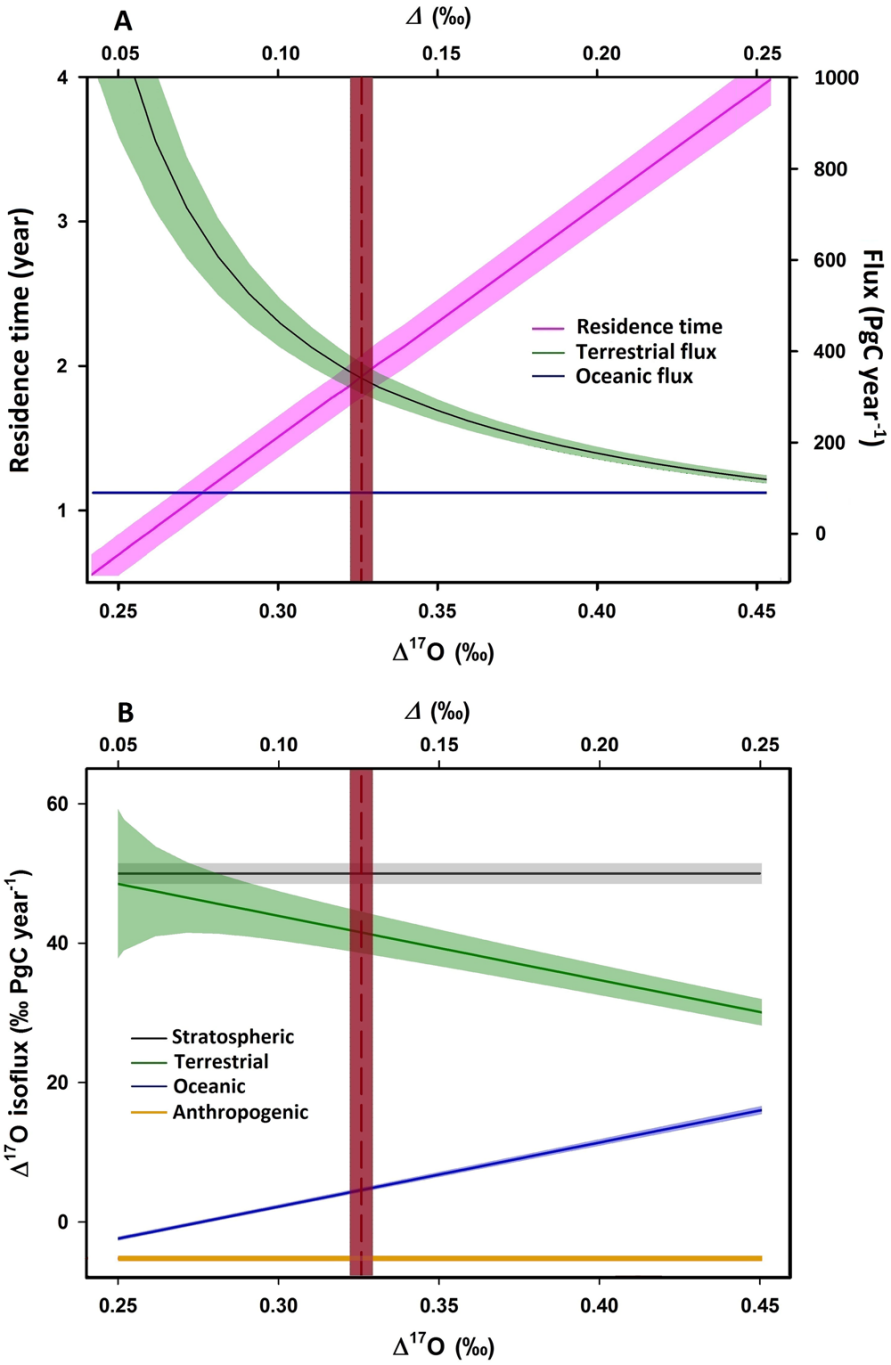
(**A**) CO_2_ recycling time (pink) as a function of Δ^17^O in atmospheric CO_2_ measured near the surface, calculated using equation (5), the box model. The corresponding oceanic (blue) and terrestrial (green) fluxes (the cycling flux between the atmosphere and terrestrial biosphere) are also shown (right-hand axis). The vertical dashed line represents the measured Δ^17^O in the tropospheric CO_2_. Shaded zones represent 1-σ error. (**B**) The isoflux of Δ^17^O from anthropogenic (gold), terrestrial (green), oceanic (blue), and stratospheric (black) sources, as a function of Δ^17^O in atmospheric CO_2_, with 1-σ error shown by the shaded areas. At Δ^17^O = 0.326‰, the terrestrial isoflux (42‰ PgC year^−1^) is a factor of 10 higher than the oceanic isoflux (5‰ PgC year^−1^), suggesting that the Δ^17^O approach has greater sensitivity to terrestrial processes than oceanic processes. For easier comparison, we have multiplied the oceanic and terrestrial isofluxes by −1.

**Figure 5:**
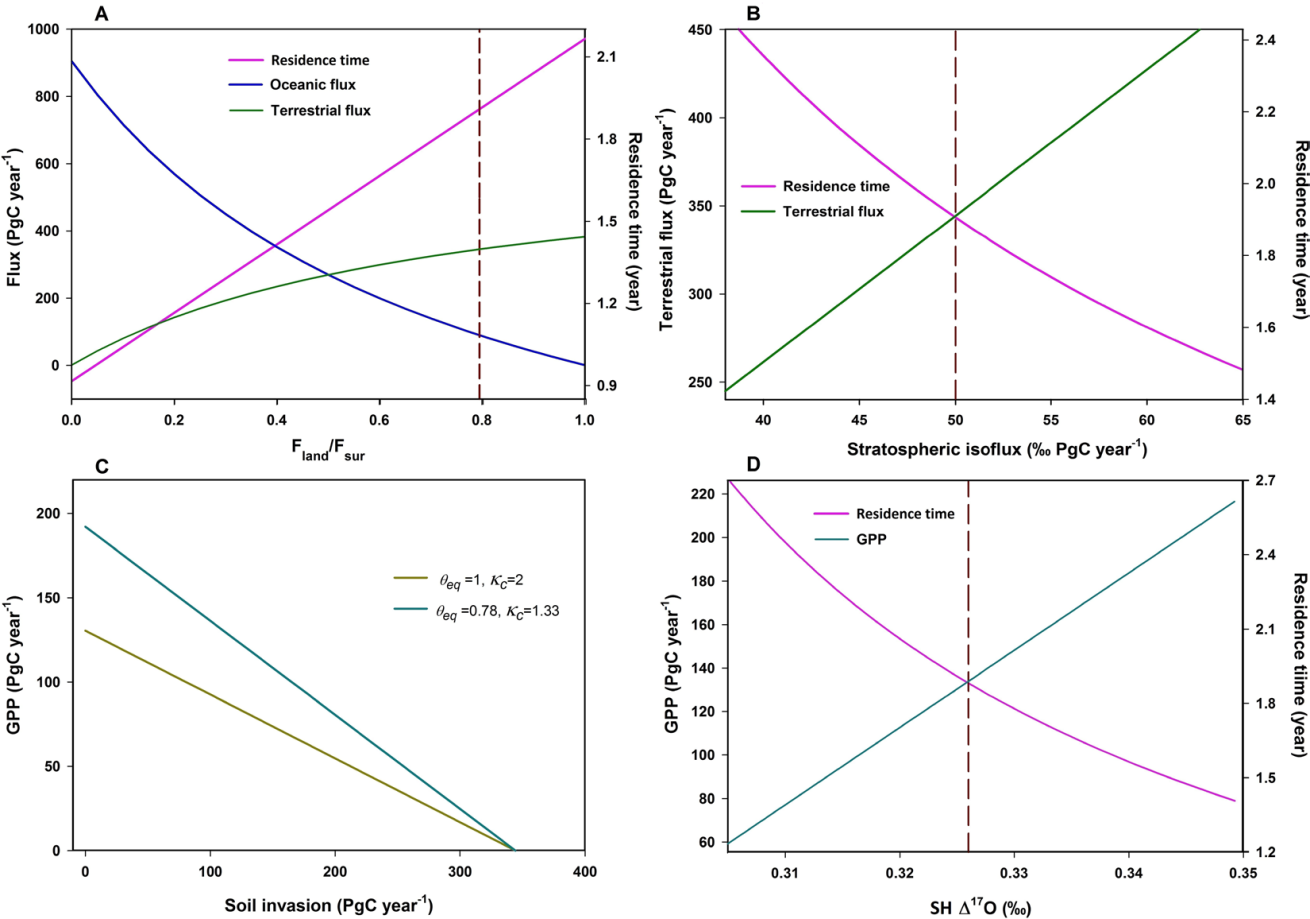
(**A**) The calculated recycling time of CO_2_ (pink line), as a function of F_land_/F_sur_. Stratospheric isoflux is adopted to be 50‰ PgC year^−1^; anthropogenic flux is 9.4 PgC year^−1^. The required fluxes from the land and ocean to reproduce the observed Δ^17^O = 0.326‰ in air CO_2_ are also shown, in green and blue, respectively. The vertical dashed line is drawn through the generally adopted oceanic flux of 90 PgC year^−1^. (**B**) The calculated recycling time of CO_2_, as a function of the stratospheric isoflux, assuming an oceanic flux of 90 PgC year^−1^ and Δ^17^O = 0.325‰ in air CO_2_. The nominal flux adopted in (**A**) is indicated by the vertical dashed line. The associated terrestrial flux is also shown. (**C**) Estimated GPP as a function of soil invasion flux, for two photosynthetic scenarios (*θ*_*eq*_ represents the degree of hydration of CO_2_ in stomata and *κ*_*c*_ is a measure of stomatal conductance). The stratospheric isoflux is set to be 50‰ PgC year^−1^, anthropogenic flux is 9.4 PgC year^−1^, and air CO_2_ Δ^17^O = 0.326‰. (**D**) Estimated GPP as a function of southern hemisphere (SH) CO_2_ Δ^17^O. The stratospheric isoflux is set to be 50‰ PgC year^−1^, anthropogenic flux is 9.4 PgC year^−1^, and northern hemisphere air CO_2_ Δ^17^O = 0.326‰ (marked by the vertical dashed line).

For stratospheric flux, global model simulations^17^ that consider various atmospheric transports yield an averaged isoflux from the stratosphere, F_st_ × (*Δ*_st_ − *Δ*_a_), of 50 ± 3‰ PgC year^−1^, consistent with that obtained and used previously (~43‰ PgC year^−1^; ref.^16,42^). Figure 4 summarizes the derived terrestrial CO_2_ flux and residence time in the atmosphere; in this particular model, the cross-hemispheric transport and mixing are not included, as the hemispheric difference in Δ^17^O was predicted to be small (<0.01‰; ref.^16^). Sensitivities of the derived quantities with respect to the variations of the relative importance of ocean flux, cross-tropopause exchange flux, soil invasion, and Δ^17^O value in the southern hemisphere are presented in Fig. 5. See below for the detail on the error assessment.

The current steady state box model is an updated version of Hoag *et al*.^16^; the major surface resetting processes are included explicitly to distinguish the terrestrial (re-)cycling fluxes from the oceanic. Previous works (Cuntz *et al*.^3^ and Hofmann *et al*.^13^) solve time-dependent equations (equation 3). In this case, kinetic fractionations becomes important, and spatiotemporal variabilities in all components of carbon/oxygen cycling and recycling fluxes are significant. As a result, spatial and temporal inhomogeneity in sampling has to be considered and evaluated critically. When more data are available, natural variability in carbon fluxes can be assessed. Therefore, for examining the global carbon flux at steady state, we take measurements spanning as much space and time as possible. For error analysis, a standard error from each component of measurements is adopted, leaving standard deviation for representing spatiotemporal variability assessment in time-dependent models.

## Assessing gross primary productivity

Plant uptake scenarios affect the estimates of GPP and soil invasion. GPP can be estimated as follows:

6 $$0.88\times {\rm{GPP}}={{\rm{F}}}_{al}-{{\rm{F}}}_{la}=\frac{{{\rm{F}}}_{{\rm{land}}}-{{\rm{F}}}_{{\rm{s}}}}{{\theta }_{eq}{\kappa }_{c}+1},$$

where *θ*_*eq*_ represents the degree of hydration of CO_2_ in stomata and *κ*_*c*_ is a measure of stomatal conductance which can be expressed by

7 $${k}_{c}={C}_{c}/({C}_{a}-{C}_{c}),$$

where *C*_*c*_ is the CO_2_ concentration in chloroplasts at the site of CO_2_ hydration and *C*_*a*_ is the atmospheric concentration. The factor 0.88 is used to account for leaf respiration^43^. For C_3_ plants, *C*_*c*_ */C*_*a*_ = 2/3; for C_4_ plants, *C*_*c*_ */C*_*a*_ = 1/3, assuming that *C*_*c*_ is equal to intracellular CO_2_ concentration^44^. A generally averaged *C*_*c*_ */C*_*a*_ is 0.57 or *κ*_*c*_ = 1.33 (ref.^8,9^). Overall, the value of *κ*_*c*_ varies between 1.33 and 2.93 (ref.^3,9^). *θ*_*eq*_ is also variable^33,45^. Careful error assessment is made to quantify the effect of this variation on the value of GPP (Fig. 5C). See Table 1 for the summary of the parameters and the associated errors and variations used in this work.

Errors resulted from incomplete understanding of sources and the Δ^17^O values in various water bodies (meteoric, soil, and oceanic waters) and global atmospheric CO_2_ (i.e., tropospheric and stratosphere-to-troposphere stream Δ^17^O). The corresponding errors are summarized in Table 1. There are, however, other related processes whose λ values remain poorly known, of which transpiration and plant uptake scenarios (i.e., chloroplast CO_2_ concentration and enzyme catalyzed oxygen exchange efficiency) are the most uncertain. Transpiration is sensitive to plant species and ambient air relative humidity^21^. If we assume an average considered in Landais *et al*.^21^, the variation in λ results in a variation of 0.017‰ for Δ^17^O for a 0.05 change in relative humidity; this term contributes ~30% of the error (~1/3 from atmospheric CO_2_ measurements and the remaining from the cross-tropopause exchange Δ^17^O flux) in estimating the global oxygen isotope residence time and terrestrial carbon flux. Proper error propagation is made to assess the error in the terrestrial flux (F_land_) derived in equation (5), followed by a Monte Carlo approach to evaluate the errors from *κ*_*c*_ and *θ*_*eq*_ in equation (6) for GPP estimate.

A function *f* with *n* independent variables is expressed as:

8 $$f=f({x}_{1},{x}_{2},{x}_{3},\ldots ,{x}_{n})$$

The error of each quantity *x*_*i*_ is given by the standard deviation (*σ*_*i*_) or standard error $(\overline{{\sigma }_{i}})$. The former refers to the error of a single measurement while the latter is used to represent the error of a sample mean. With a large set of measurements, we use $\overline{{\sigma }_{i}}$ to represent the error of the sample mean $\overline{{x}_{i}}$. The errors in equation (4) refer to such an error. Following standard error propagation, the error of the function *f* is

9 $${\overline{{\sigma }_{f}}}^{2}=\,\sum _{i=1}^{i=n}{(\frac{\partial f}{\partial {x}_{i}})}^{2}{\overline{{\sigma }_{i}}}^{2}$$

For a single measurement, i.e., one measurement only for each of the variable *x*_*i*_, the final error *σ*_*f*_ is the square root of $\sum _{i=1}^{i=n}{(\frac{\partial f}{\partial {x}_{i}})}^{2}{\sigma }_{i}^{2}.\,$ Therefore, for a set of many measurements reported in this work, the estimated terrestrial flux F_land_ is 345 PgC year^−1^, with 1 standard error being 70 PgC year^−1^. However, for each of the variables in equation (4) (Δ^17^_a_, Δ^17^_l_ or Δ^17^O_mw_, F_oa_, F_anth_, and F_st_ × (Δ^17^_st_ − Δ^17^_a_)), if we measure them once, the error, or sample inhomogeneity due to inhomogeneity in sources and processes, increases to 600 PgC year^−1^. For errors resulting from global carbon cycling scenarios, a Monte Carlo simulation is employed. The final error is estimated from 500 × 500 × 500 = 125 M (500 samplings for the terrestrial flux and 500 each for two variables in the carbon model in equation (6); the errors are converged with >300 random samplings) calculations of the model using repeated random sampling of each of the three variables. From the literature, *κ*_*c*_ ranges from 1.33 to 2.97 (depending critically on *C*_*c*_; ref.^3,9^) and *θ*_*eq*_ from 0.7 to 0.78 (affected by enzyme carbonic anhydrase activity in C_4_ plants; ref.^9,33,44^). To consider final total errors in GPP and F_s_, we perform Monte Carlo simulations to randomly determine values for *κ*_*c*_ and *θ*_*eq*_ over the aforementioned ranges. The distribution of these two parameters is assumed to be uniform, with the measurement error of Δ^17^O assumed to be following normal distribution.

## Oxygen isotope residence time and gross primary productivity

Figure 4 summarizes the model results calculated using equation (5) and a value for Δ^17^O of 0.326 ± 0.005‰ for the troposphere, the average for the four locations discussed above. The current mass loading of atmospheric CO_2_ (*M*) is 828 ± 10 PgC (ref.^1,45^). The CO_2_ oxygen isotope residence time τ is given by *M*/F_sur_, where F_sur_ = F_land_ + F_ao_. Taking into account the aforementioned uncertainties of the parameters in equation (5), the terrestrial flux F_land_ is determined to be 345 ± 70 PgC year^−1^, and τ is 1.9 ± 0.3 years, consistent with previous estimates^2,3,8,9^. The estimate is insensitive to the partitioning between ocean and terrestrial fluxes because of the sensitivity of the Δ^17^O approach to the terrestrial processes (Fig. 4). However, we show below that we cannot constrain the value for GPP better than other methods, because of unknown quantities for soil invasion and degree of isotopic equilibrium between leaf water and stomatal CO_2_.

No significant advancement toward quantifying soil invasion has been made since Wingate *et al*.^7^. The reported flux can be as low as <10 PgC year^−1^ (ref.^6^) to as high as 450 PgC year^−1^ (ref.^7^), depending on the catalyzed hydration activity (via enzyme carbonic anhydrase). Recently, the hydration activity has been found to likely be high, resulting in an invasion flux as high as respiration^7^, and we choose this as our best estimate, i.e., F_s_ = F_r_. By definition, GPP is the sum of respiration F_r_ and NEP (net ecosystem productivity). So equation (6) can be expressed by

10 $${\rm{GPP}}\,={0.88}^{-1}\times \frac{{{\rm{F}}}_{{\rm{land}}}-{{\rm{F}}}_{{\rm{s}}}}{{\theta }_{eq}{\kappa }_{c}+1}={\rm{NEP}}+{{\rm{F}}}_{{\rm{r}}}={\rm{NEP}}+{{\rm{F}}}_{{\rm{s}}}.$$

The globally estimated NEP is 10 PgC year^−1^ (ref.^46^). Once the values of *κ*_*c*_ and *θ*_*eq*_ are chosen, along with the value of F_land_ reported above, GPP and F_s_ can be derived.

With a previously suggested plant uptake scenario (*κ*_*c*_ = 1.33 and *θ*_*eq*_ = 0.78; ref.^8^) and an independent constraint for net ecosystem productivity^46,47^, we derive an estimate of 130 ± 25 PgC year^−1^ for GPP, with a best guess for soil invasion of 120 ± 20 PgC year^−1^ (calculated from equation (10)). A Monte Carlo simulation that considers various carbon cycling models^3,8,9,33,44^, including plant types and degree of oxygen equilibrium with various water bodies in the biosphere and hydrosphere, gives the estimates of GPP and soil invasion to 120 ± 30 and 110 ± 30 PgC year^−1^, respectively. The estimated GPP is toward the lower end of Welp *et al*.^8^ but close to that of Beer *et al*.^4^. Given that the value of Δ^17^O is sensitive to the isofluxes between atmospheric CO_2_ and water bodies, we expect that extended studies with multiple CO_2_ isotopologues into C_3_-dominated regions such as the Amazonian rainforest, C_4_-dominated lands such as grasslands in North America, and vegetation-sparse areas such as the Canadian arctic could have great potential to refine the partitioning between photosynthesis, respiration and soil invasion, and thus to provide a better estimate of the terrestrial GPP.

Figure 5 shows the sensitivities of the derived quantities with respect to the variations of the ocean flux (expressed as variations in the fraction of flux from land to flux from the total surface) (A), cross-tropopause exchange flux (B), soil invasion (C), and Δ^17^O value in the southern hemisphere (D). Because of the low value of the oceanic isoflux shown in Fig. 4B, the ocean affects the derived terrestrial flux and CO_2_ residence time weakly; changing the oceanic flux by 50% changes the residence time by 0.1 year. The stratospheric flux, however, is more sensitive. 10% changes in the stratospheric flux result in ~0.2 year changes in the residence time. The sensitivity to soil invasion (Fig. 5C) is calculated by varying the variable with F_land_ fixed at 345 PgC year^−1^. Depending on soil invasion and plant uptake scenarios (the values of *κ*_*c*_ and *θ*_*eq*_), the value of GPP could vary between 0 and 200 PgC year^−1^. Another important value that remains undetermined is the value of Δ^17^O of tropospheric CO_2_ in the southern hemisphere. Figure 5D shows that the GPP and residence time are sensitive to the Δ^17^O value in the southern hemisphere; the sensitivity is obtained by assuming F_s_ = 110 PgC year^−1^, F_land_ = 345 PgC year^−1^, *κ*_*c*_ = 1.33, and *θ*_*eq*_ = 0.78. If Δ^17^O in the southern hemisphere is 0.01‰ (0.02‰) higher than that in the northern hemisphere, the GPP value increases to 170 (200) PgC year^−1^; the residence time then reduces to 1.7 (1.5) years. As soon as the value of Δ^17^O in the southern hemisphere is measured, the global residence time can be determined and GPP can be better quantified.

In summary, the triple-oxygen isotopic composition of CO_2_ constrains the global oxygen isotopic residence time in the atmosphere to 1.9 ± 0.3 years, compared to 0.9–1.7 years (ref.^8^) or longer^2,3,9^ reported previously. The terrestrial flux is quantified to be 345 ± 70 PgC year^−1^, falling in the range reported in the literature, 200–660 PgC year^−1^ (ref.^2,3,8,9^). Because of the isotope recycling time of CO_2_, the spatial inhomogeneity of Δ^17^O obtained between localities shows that the commonly used δ values can be applied to Δ^17^O to refine knowledge of the flux partitioned between respiration/soil invasion, photosynthesis, and air-sea exchange. CO_2_ sampling campaigns in the remote Pacific and southern hemisphere oceans can better remove interference from terrestrial processes, to quantify the oceanic flux. High-resolution global and regional models assimilating CO_2_ isotopologues (Δ^17^O in particular) with online carbon and water cycle modules can potentially strengthen our understanding of the associated processes at molecular scales. We expect that existing models^3^ coupled with a cross-tropopause exchange module extending surface biogeochemical models to include stratospheric processes will greatly improve our estimates and provide extraordinary precision to probe the associated fluxes in the global carbon and water cycles involving CO_2_.

## Electronic supplementary material

Supplementary Information

## References

[1] Stocker, T. F., Dahe, Q., & Plattner, G. K. Climate Change 2013: The Physical Science Basis, Working Group I Contribution to the Fifth Assessment Report of the Intergovernmental Panel on Climate Change. Summary for Policymakers (IPCC, 2013).

[2] Ciais P (1997). A three-dimensional synthesis study of δ^18^O in atmospheric CO_2_. 1. Surface fluxes. Journal of Geophysical Research-Atmospheres.

[3] Cuntz M (2003). A comprehensive global three-dimensional model of δ^18^O in atmospheric CO_2_: 2. Mapping the atmospheric signal. J. Geophys. Res..

[4] Beer C (2010). Terrestrial Gross Carbon Dioxide Uptake: Global Distribution and Covariation with Climate. Science.

[5] Francey RJ, Tans PP (1987). Latitudinal variation in O^18^ of atmospheric CO_2_. Nature.

[6] Stern LA, Amundson R, Baisden WT (2001). Influence of soils on oxygen isotope ratio of atmospheric CO_2_. Global Biogeochemical Cycles.

[7] Wingate L (2009). The impact of soil microorganisms on the global budget of δ^18^O in atmospheric CO_2_. Proceedings of the National Academy of Sciences of the United States of America.

[8] Welp LP (2011). Interannual variability in the oxygen isotopes of atmospheric CO_2_ driven by El Niño. Nature.

[9] Farquhar GD (1993). Vegetation effects on the isotope composition of oxygen in atmospheric CO_2_. Nature.

[10] Liang MC, Mahata S, Laskar AH, Bhattacharya SK (2017). Spatiotemporal Variability of Oxygen Isotope Anomaly in near Surface Air CO_2_ over Urban, Semi-Urban and Ocean Areas in and around Taiwan. Aerosol and Air Quality Research.

[11] Barkan E, Luz B (2012). High‐precision measurements of ^17^O/^16^O and ^18^O/^16^O ratios in CO_2_. Rapid communications in mass spectrometry.

[12] Thiemens MH, Chakraborty S, Jackson TL (2014). Decadal Δ^17^O record of tropospheric CO_2_: Verification of a stratospheric component in the troposphere. Journal of Geophysical Research-Atmospheres.

[13] Hofmann MEG, Horváth B, Schneider L, Peters W, Schützenmeister K, Pack A (2017). Atmospheric measurements of Δ^17^O in CO_2_ in Göttingen, Germany reveal a seasonal cycle driven by biospheric uptake. Geochimica et Cosmochimica Acta.

[14] Jacob DJ, Prather MJ, Wofsy SC, McElroy MB (1987). Atmospheric distribution of ^85^Kr simulated with a general circulation model. Journal of Geophysical Research.

[15] Lal D (1966). & Rama, Characteristics of global tropospheric mixing based on man-made C^14^, H^3^, and Sr^90^. Journal of Geophysical Research.

[16] Hoag K, Still C, Fung I, Boering KA (2005). Triple oxygen isotope composition of tropospheric carbon dioxide as a tracer of terrestrial gross carbon fluxe. Geophys. Res. Lett..

[17] Liang, M. C., Blake, G. A. & Yung, Y. L. Seasonal cycle of C^16^O^16^O, C^16^O^17^O, and C^16^O^18^O in the middle atmosphere: Implications for mesospheric dynamics and biogeochemical sources and sinks of CO_2_. *Journal of Geophysical Research-Atmospheres* **113**, 10.1029/2007jd008392 (2008).

[18] Liang MC, Mahata S (2015). Oxygen anomaly in near surface carbon dioxide reveals deep stratospheric intrusion. Scientific Reports.

[19] Laskar AH, Mahata S, Liang MC (2016). Identification of anthropogenic CO_2_ using triple oxygen and clumped isotopes. Environmental Science & Technology.

[20] Luz B, Barkan E (2010). Variations of ^17^O/^16^O and ^18^O/^16^O in meteoric waters. Geochimica et Cosmochimica Acta.

[21] Landais A, Barkan E, Yakir D, Luz B (2006). The triple isotopic composition of oxygen in leaf water. Geochimica et Cosmochimica Acta.

[22] Barkan E, Luz B (2007). Diffusivity fractionations of H_2_^16^O/H_2_^17^O and H_2_^16^O/H_2_^18^O in air and their implications for isotope hydrology. Rapid Communications in Mass Spectrometry.

[23] Hofmann MEG, Horvath B, Pack A (2012). Triple oxygen isotope equilibrium fractionation between carbon dioxide and water. Earth and Planetary Science Letters.

[24] Luz B, Barkan E (2005). The isotopic ratios ^17^O/^16^O and ^18^O/^16^O in molecular oxygen and their significance in biogeochemistry. Geochimica et Cosmochimica Acta.

[25] Luz B, Barkan E, Bender ML, Thiemens MH, Boering KA (1999). Triple-isotope composition of atmospheric oxygen as a tracer of biosphere productivity. Nature.

[26] Cao X, Liu Y (2011). Equilibrium mass-dependent fractionation relationships for triple oxygen isotopes. Geochimica et Cosmochimica Acta.

[27] Luz B, Barkan E (2000). Assessment of oceanic productivity with the triple-isotope composition of dissolved oxygen. Science.

[28] Jurikova H, Guha T, Abe O, Shiah FK, Wang CH, Liang MC (2016). Variations in triple isotope composition of dissolved oxygen and primary production in a subtropical reservoir. Biogeosciences.

[29] Thiemens MH, Jackson T, Zipf EC, Erdman PW, Vanegmond C (1995). Carbon-dioxide and oxygen-isotope anomalies in the mesosphere and stratosphere. Science.

[30] Liang MC, Blake GA, Lewis BR, Yung YL (2007). Oxygen isotopic composition of carbon dioxide in the middle atmosphere. Proceedings of the National Academy of Sciences of the United States of America.

[31] Thiemens MH, Jackson T, Mauersberger K, Schueler B, Morton J (1991). Oxygen isotope fractionation in stratospheric CO_2_. Geophysical Research Letters.

[32] Yung YL, DeMore WB, Pinto JP (1991). Isotopic exchange between carbon-dioxide and ozone via O(^1^D) in the stratosphere. Geophysical Research Letters.

[33] Gillon J, Yakir D (2001). Influence of carbonic anhydrase activity in terrestrial vegetation on the ^18^O content of atmospheric CO_2_. Science.

[34] Dai A (2006). Recent Climatology, Variability, and Trends in Global Surface Humidity. Journal of Climate.

[35] Brenninkmeijer CAM, Kraft P, Mook WG (1983). Oxygen isotope fractionation between CO_2_ and H_2_O. Isotope Geoscience.

[36] Mahata S, Bhattacharya SK, Wang CH, Liang MC (2013). Oxygen isotope exchange between O_2_ and CO_2_ over hot platinum: An innovative technique for measuring Δ^17^O in CO_2_. Anal. Chem..

[37] Mahata S, Bhattacharya SK, Liang MC (2016). An improved method of high‐precision determination of Δ^17^O of CO_2_ by catalyzed exchange with O_2_ using hot platinum. Rapid Communications in Mass Spectrometry.

[38] Newman, S. *et al*. Changes in mixing ratio and isotopic composition of CO_2_ in urban air from the Los Angeles basin, California, between 1972 and 2003. *Journal of Geophysical Research-Atmospheres* **113**, doi:10.1029/2008JD00999 (2008).

[39] Gemery PA, Trolier M, White JW (1996). Oxygen isotope exchange between carbon dioxide and water following atmospheric sampling using glass flasks. Journal of Geophysical Research: Atmospheres.

[40] Solomon, S. Climate change 2007-the physical science basis: Working group I contribution to the fourth assessment report of the IPCC, Cambridge University Press (2007).

[41] Peters GP, Marland G, Le Quéré C, Boden T, Canadell JG, Raupach MR (2012). Rapid growth in CO_2_ emissions after the 2008-2009 global financial crisis. Nature Climate Change.

[42] Boering KA (2004). Observations of the anomalous oxygen isotopic composition of carbon dioxide in the lower stratosphere and the flux of the anomaly to the troposphere. Geophysical Research Letters.

[43] Pearcy R, Ehleringer J (1984). Comparative ecophysiology of C_3_ and C_4_*plants*. Plant, Cell & Environment.

[44] Cousins AB, Badger MR, von Caemmerer S (2008). C_4_ photosynthetic isotope exchange in NAD-ME- and NADP-ME-type grasses. Journal of Experimental Botany.

[45] Joos F (2013). Carbon dioxide and climate impulse response functions for the computation of greenhouse gas metrics: a multi-model analysis. Atmospheric Chemistry and Physics.

[46] Saugier, B., Roy, J. & Mooney, H. A. Estimations of global terrestrial productivity: converging toward a single number. *Terrestrial global productivity*, 543–557 (Academic Press, 2001).

[47] Houghton, J. T. *et al*. Climate change 2001: the scientific basis. (2001).

[48] Li S, Levin NE, Chesson LA (2015). Continental scale variation in ^17^O-excess of meteoric waters in the United States. Geochimica et Cosmochimica Acta.

[49] Landais A, Risi C, Bony S, Vimeux F, Descroix L, Falourd S, Bouygues A (2010). Combined measurements of ^17^O_excess_ and d-excess in African monsoon precipitation: Implications for evaluating convective parameterizations. Earth and Planetary Science Letters.

[50] Miller JB, Yakir D, White JWC, Tans PP (1999). Measurement of ^18^O/^16^O in the soil-atmosphere CO_2_ flux. Global Biogeochemical Cycles.

[51] Vogel JC, Grootes PM, Mook WG (1970). Isotopic fractionation between gaseous and dissolved carbon dioxide. Zeitschrift Fur Physik.

