# Supplementary material for: Oxygen isotope anomaly in tropospheric CO2 and implications for CO2 residence time in the atmosphere and gross primary productivity
Source: Sci Rep. 2017 Oct 13;7:13180. doi: 10.1038/s41598-017-12774-w (PMC5640618; doi:10.1038/s41598-017-12774-w)
Supplement: Supplementary file 1 — Supplementary Information [file 41598_2017_12774_MOESM1_ESM.pdf]

Supplementary Information

Oxygen isotope anomaly in tropospheric CO_2 and implications for CO_2 residence time in the atmosphere and gross primary productivity

Mao-Chang Liang[1,2], Sasadhar Mahata[1], Amzad H. Laskar[1], Mark H. Thiemens[3], Sally Newman[4]

[1]Research Center for Environmental Changes, Academia Sinica, Taipei, Taiwan

[2]Graduate Institute of Astronomy, National Central University, Taipei, Taiwan

[3]Department of Chemistry and Biochemistry, University of California at San Diego, La Jolla, USA

[4]Division of Geological and Planetary Sciences, California Institute of Technology, Pasadena, USA

Table S1: Summary of CO_2 data collected at Academia Sinica (AS), National Taiwan University (NTU), South China Sea (SCS), and Palos Verdes (California, USA; PVD). Taipei=AS+NTU. Values of $\delta^{13}C$ and $\delta^{18}O$ are referenced to V-PDB and V-SMOW, respectively.

Sampling date & time	$[CO_2]$ (ppmv)	$\delta^{13}C$ (‰)	$\delta^{18}O$ (‰)	$\Delta^{17}O$ (‰)	Note(s)
Academia Sinica (AS)					
2012/12/28 11:45	N/A	-8.52	41.50	0.230	Latitude:
17:00	N/A	-8.82	41.48	0.330	25°02'27" N
2013/01/02 10:15	N/A	-9.21	39.09	0.270	
2013/01/15 11:00	N/A	-8.49	39.41	0.350	Longitude:
2013/01/24 11:00	365.2	-8.77	41.68	0.258	121°36'51" E
12:52	398.6	-9.12	40.91	0.238	
19:00	475.1	-9.87	39.84	0.345	Elevation:
2013/09/24 10:40	392.8	-8.11	41.25	0.256	60 m a.s.l.
14:10	392.3	-8.08	41.17	0.322	
20:00	408.7	-8.86	40.70	0.294	
2013/09/25 14:30	398.4	-8.38	40.96	0.352	
23:30	402.5	-8.61	40.12	0.328	
2013/10/07 11:30	404.7	-8.62	40.47	0.252	
14:50	397.0	-8.26	41.14	0.216	
22:20	462.6	-11.08	39.27	0.264	
2013/10/08 10:20	432.6	-9.83	39.62	0.294	
14:40	424.4	-9.48	40.02	0.245	
21:40	425.1	-9.52	40.25	0.292	
2013/10/16 10:30	403.4	-8.67	40.49	0.301	
14:20	404.4	-8.64	40.48	0.269	
21:00	420.4	-9.36	40.23	0.290	
2013/10/17 10:00	399.8	-8.37	40.76	0.322	
14:30	402.3	-8.05	40.23	0.315	
15:00	402.1	-8.51	40.36	0.306	
17:20	409.3	-8.44	39.89	0.325	
23:59	434.5	-9.78	39.77	0.335	
2013/10/25 10:00	411.0	-8.76	40.39	0.335	
14:05	411.1	-8.81	40.45	0.294	
23:45	415.2	-9.00	40.15	0.313	
2013/10/26 10:00	410.6	-8.85	40.40	0.307	
2013/10/27 00:45	430.6	-9.62	40.26	0.312	
2013/10/30 10:10	N/A	-7.34	40.42	0.368	
14:30	394.6	-6.96	40.51	0.415	
2013/10/31 10:00	399.0	-8.39	41.22	0.280	
14:00	397.8	-8.35	41.20	0.327	
22:20	406.0	-8.69	41.02	0.330	
2013/11/04 10:30	411.1	-8.78	40.49	0.309	

	14:30	405.9	-8.64	40.60	0.324	
	18:30	414.9	-9.02	40.37	0.347	
2013/11/09	10:30	405.1	-8.34	41.08	0.334	
	18:30	425.3	-9.43	40.30	0.356	
2013/11/19	10:00	419.6	-8.74	40.58	0.371	
	14:00	418.4	-8.71	40.50	0.358	
	18:00	414.6	-8.91	40.54	0.337	
2013/11/26	11:00	409.7	-8.47	40.74	0.336	
	15:00	406.5	-8.52	40.86	0.313	
	21:00	N/A	-9.61	40.28	0.291	
2014/01/27	10:30	403.0	-8.52	41.31	0.388	
	15:20	400.0	-8.68	41.21	0.383	
	18:00	404.5	-8.64	41.31	0.390	
2014/02/03	11:10	408.8	-8.80	41.18	0.370	
	14:30	409.0	-8.86	41.38	0.353	
	19:30	409.0	-8.95	41.40	0.371	
	19:30	409.0	-8.95	41.40	0.376	
2014/02/17	10:30	445.9	-10.30	40.39	0.291	
	14:30	408.6	-8.74	41.51	0.354	
	18:00	437.0	-9.92	41.06	0.340	
2014/02/19	10:00	418.0	-9.12	40.59	0.329	
	18:00	424.0	-9.38	40.39	0.373	
	15:35	403.2	-8.55	40.36	0.306	
2014/02/20	14:30	410.6	-8.81	40.94	0.355	
	18:00	417.2	-9.02	40.65	0.301	
2014/02/22	12:15	401.0	-8.44	41.47	0.400	
	17:00	402.0	-8.36	41.49	0.349	
2014/02/24	17:30	406.6	-8.63	41.56	0.397	
2014/03/06	16:30	N/A	-10.18	39.28	0.267	
2014/04/16	14:40	413.9	-8.75	40.01	0.272	
2014/05/08	10:42	471.7	-10.67	40.26	0.330	
2014/06/04	10:40	423.5	-9.30	40.83	0.376	
	12:30	420.2	-9.17	41.17	0.376	
	17:00	417.3	-9.17	41.13	0.408	
2014/06/14	19:45	415.6	-9.13	39.92	0.349	
2014/07/21	10:50	402.8	-8.40	41.41	0.354	
2014/07/22	09:45	405.0	-8.63	41.19	0.372	
2014/07/24	10:10	423.0	-9.42	40.41	0.378	
2014/07/25	10:00	457.3	-10.57	39.19	0.334	
	11:00	412.5	-8.91	40.08	0.383	
	15:15	433.4	-9.53	40.05	0.364	
2014/08/05	10:20	420.4	-9.18	37.33	0.302	
2014/08/06	10:25	420.7	-9.31	39.61	0.374	
2014/08/07	11:50	422.6	-9.56	40.22	0.375	
2014/08/08	11:20	416.9	-9.24	39.96	0.390	

2014/09/03 09:00	455.2	-10.58	39.55	0.420	
12:00	431.5	-9.64	41.96	0.262	
2014/10/27 11:10	408.4	-8.50	41.34	0.324	
14:23	403.7	-8.60	41.25	0.299	
18:01	416.7	-8.84	41.10	0.317	
2014/11/18 09:30	414.1	-9.20	40.49	0.305	
14:00	412.2	-9.01	40.64	0.360	
18:08	415.2	-9.04	40.57	0.362	
2015/01/16 10:00	412.7	-8.20	41.25	0.326	
13:00	412.3	-8.82	41.17	0.343	
2015/01/26 14:00	421.1	-9.02	41.29	0.334	
2015/03/19 13:20	420.7	-8.83	41.80	0.355	
13:30	421.6	-8.86	41.76	0.352	
13:40	424.5	-8.97	41.66	0.341	
13:50	420.8	-8.78	41.80	0.359	
14:00	420.1	-8.75	41.75	0.365	
2015/04/02 09:30	453.6	-10.54	40.61	0.330	
12:53	450.2	-10.11	40.85	0.305	
18:30	423.9	-9.39	41.42	0.299	
2015/04/23 10:20	405.9	-8.90	41.56	0.296	
14:30	405.3	-8.99	41.67	0.342	
2015/05/08 09:25	407.6	-7.97	40.14	0.312	
12:40	423.7	-9.25	40.87	0.309	
17:30	452.5	-10.54	40.42	0.346	
2015/05/11 14:30	402.7	-8.41	42.05	0.348	
17:30	410.2	-9.36	41.04	0.300	
2015/05/28 09:00	423.5	-9.60	40.51	0.261	
13:20	399.8	-8.90	41.33	0.321	
17:52	415.5	-9.30	41.24	0.331	
2015/06/11 09:50	423.5	-9.56	40.76	0.328	
13:05	399.8	-9.05	41.71	0.331	
17:15	415.5	-10.44	40.78	0.330	
2015/06/25 09:20	417.3	-9.57	40.17	0.320	
13:10	411.0	-9.53	40.66	0.334	
17:15	423.5	-9.71	40.20	0.346	
2015/07/23 09:40	N/A	-9.28	41.08	0.374	
13:00	404.9	-8.94	41.35	0.379	
17:00	427.5	-9.69	39.96	0.327	
2015/08/06 09:20	412.3	-9.13	40.93	0.403	
13:10	401.4	-8.63	41.48	0.368	
17:00	400.7	-9.26	41.59	0.375	
2015/08/20 09:25	398.3	-8.56	41.68	0.380	
13:10	397.3	-8.59	41.66	0.382	
17:50	399.7	-9.61	39.72	0.397	
2015/09/03 09:30	425.8	-9.84	39.74	0.335	

12:45	418.4	-9.56	40.48	0.363	
17:30	422.1	-10.12	39.30	0.398	
2015/09/15 13:30	403.2	-8.69	41.12	0.397	
17:00	408.8	-8.70	40.65	0.391	
2015/10/13 09:30	399.5	-8.85	40.62	0.421	
13:20	400.7	-8.86	40.80	0.357	
2015/10/27 09:30	435.6	-10.17	40.03	0.328	
13:30	416.9	-9.42	40.73	0.347	
2015/11/13 09:15	435.8	-10.28	39.82	0.340	
13:00	409.4	-9.12	40.64	0.347	
17:00	443.1	-10.43	39.45	0.331	
2015/11/27 09:30	407.6	-8.88	40.81	0.332	
13:30	406.8	-8.93	40.91	0.340	
17:00	411.8	-9.04	40.92	0.374	
2015/12/08 09:15	407.3	-8.96	40.71	0.330	
17:15	409.2	-9.12	40.78	0.347	
2015/12/22 09:30	407.4	-9.56	40.63	0.330	
13:30	391.1	-8.79	41.46	0.403	
17:00	392.7	-8.86	41.37	0.324	
**Average**	**414.6**	**-9.07**	**40.70**	**0.336**	**n = 146**
**Stdev**	**16.2**	**0.65**	**0.71**	**0.041**	
National Taiwan University (NTU)					
2013/11/14 10:10	353.8	-7.95	40.94	0.354	Latitude:
14:03	366.2	-8.02	41.29	0.358	25°00'53" N
19:20	462.1	-9.94	38.32	0.375	
2013/11/15 10:40	416.1	-9.12	39.41	0.414	Longitude:
14:05	421.1	-9.19	39.34	0.382	121°32'21" E
19:12	438.3	-9.92	38.26	0.335	
2013/11/16 10:55	412.2	-8.78	40.02	0.324	Elevation:
14:22	408.4	-8.70	40.24	0.324	20 m a.s.l.
19:28	462.1	-7.27	38.52	0.331	
2013/11/24 11:07	365.2	-8.77	41.68	0.258	
12:52	398.6	-9.12	40.91	0.238	
19:00	475.1	-9.87	39.84	0.345	
2013/11/28 11:20	416.3	-9.29	40.04	0.294	
14:32	421.5	-9.18	40.25	0.315	
20:27	N/A	-9.60	40.20	0.399	
2013/12/01 10:53	408.2	-8.81	40.72	0.296	
14:03	N/A	-8.91	40.61	0.315	
15:42	411.6	-8.96	40.60	0.349	
2013/12/07 12:07	404.2	-8.56	41.34	0.311	
16:16	409.9	-8.63	41.18	0.339	
2014/01/07 15:12	N/A	-9.90	40.71	0.333	
22:27	N/A	-10.72	39.92	0.331	
2014/01/20 11:42	436.8	-9.93	40.31	0.345	

15:00	420.2	-9.15	40.72	0.340	
22:15	425.5	-9.52	40.47	0.406	
2014/04/17 07:05	447.5	-10.15	40.73	0.360	
09:05	429.0	-9.94	40.85	0.367	
10:05	424.4	-9.45	41.21	0.339	
12:00	415.6	-9.39	41.49	0.363	
13:12	414.3	-9.24	41.25	0.349	
15:07	429.8	-10.26	40.79	0.356	
17:10	434.7	-9.62	40.82	0.391	
2014/07/22 10:10	408.8	-8.70	41.11	0.270	
14:10	419.1	-9.72	40.92	0.370	
19:15	411.7	-8.99	41.10	0.427	
2014/07/23 15:45	438.3	-9.79	37.68	0.281	
2014/07/25 11:15	408.3	-8.87	40.86	0.285	
22:10	476.7	-11.42	37.84	0.332	
2014/07/26 01:12	454.7	-10.11	39.28	0.298	
2014/09/03 09:00	444.9	-10.40	39.71	0.335	
12:00	424.0	-9.29	40.46	0.333	
15:00	413.8	-8.88	41.15	0.328	
2015/03/19 10:26	436.7	-10.79	40.73	0.318	
13:13	420.7	-9.28	41.49	0.332	
13:21	421.2	-9.38	41.36	0.325	
13:30	421.6	-9.07	41.59	0.373	
13:39	424.5	-9.13	41.56	0.336	
13:50	420.8	-9.20	41.86	0.345	
14:00	420.1	-9.06	41.60	0.356	
17:00	413.8	-9.02	41.64	0.323	
2015/04/23 14:00	423.3	-9.99	41.25	0.347	
17:15	423.3	-9.99	41.25	0.355	
2015/05/08 17:15	478.4	-11.11	40.41	0.341	
2015/05/11 09:00	428.2	-10.27	40.60	0.298	
13:20	428.2	-8.81	42.06	0.287	
16:51	406.5	-9.18	41.63	0.326	
2015/05/28 08:43	428.9	-10.16	40.48	0.290	
13:10	398.0	-8.59	42.09	0.296	
17:45	420.7	-9.27	41.19	0.333	
2015/06/25 10:14	415.1	-9.36	40.46	0.359	
13:13	408.1	-9.17	41.14	0.365	
17:09	414.6	-9.12	41.33	0.329	
2015/07/23 09:00	N/A	-15.19	38.57	0.304	
17:00	N/A	-13.12	38.78	0.257	
2015/08/06 13:00	N/A	-9.09	41.06	0.352	
2015/08/20 09:15	398.3	-8.82	41.92	0.354	
0 13:00	398.4	-8.60	40.76	0.309	
17:00	400.1	-8.75	40.82	0.283	

2015/09/15 09:00	417.4	-9.46	40.29	0.311	
13:00	404.6	-8.86	40.62	0.337	
17:00	417.4	-9.14	40.42	0.332	
2015/10/13 09:10	399.5	-8.92	40.67	0.320	
13:15	404.6	-8.88	40.67	0.359	
17:00	396.8	-9.07	40.62	0.330	
2015/10/27 09:00	451.1	-10.74	39.68	0.339	
13:20	415.4	-9.51	40.47	0.317	
17:00	415.5	-9.38	40.66	0.373	
2015/11/13 09:05	439.2	-10.26	39.76	0.336	
13:20	415.6	-9.96	40.03	0.344	
17:05	431.4	-9.40	40.35	0.372	
2015/11/27 09:15	409.6	-9.10	40.76	0.405	
13:15	409.5	-8.97	40.96	0.340	
17:30	416.6	-9.18	40.90	0.366	
2015/12/08 09:20	407.7	-8.94	41.07	0.334	
13:13	404.4	-8.44	41.82	0.352	
17:00	408.8	-9.12	40.98	0.325	
2015/12/22 09:20	N/A	-11.32	39.58	0.306	
13:20	396.6	-8.78	41.71	0.340	
17:00	395.8	-8.88	37.47	0.276	
**Average**	**419.0**	**-9.46**	**40.56**	**0.335**	**n = 89**
**Stdev**	**21.4**	**1.02**	**0.98**	**0.035**	
**Average(Taipei)**	**416.2**	**-9.22**	**40.65**	**0.335**	**n = 235**
**Stdev(Taipei)**	**18.3**	**0.83**	**0.82**	**0.039**	
South China Sea (SCS)					
2013/06/18 18:35	N/A	-8.27	41.81	0.313	18°18'14" N 118°17'23" E
2013/06/21 14:35	N/A	-8.32	41.91	0.365	11°47'00' N 113°45'00' E
2013/06/22 13:45	N/A	-8.98	41.29	0.296	10°32'00' N 114°22'00" E
2013/06/26 18:44	N/A	-8.43	41.90	0.383	13°16'19" N 116°16'36" E
2013/06/27 08:35	N/A	-8.48	42.12	0.371	15°07'17" N 116°59'35" E
2013/06/28 08:37	N/A	-8.67	41.65	0.328	19°38'26" N 118°0'56" E
2013/10/15 08:15	403.2	-8.42	40.84	0.332	21°17'15" N 118°57'47" E
13:15	400.8	-8.46	40.79	0.301	20°48'52" N 118°31'56" E
18:00	406.1	-8.75	40.53	0.313	20°15'05" N 118°01'19" E
2013/10/16 07:00	391.6	-8.76	40.52	0.294	18°41'15" N

					116°36'36" E
12:05	397.8	-8.44	40.85	0.329	18°03'14" N<br>116°02'46" E
14:00	391.7	-8.30	40.95	0.400	18°03'14" N<br>116°02'46" E
17:20	395.2	-8.31	41.01	0.346	18°03'14" N<br>116°02'46" E
2013/10/17 08:40	383.5	-8.26	40.40	0.317	18°03'14" N<br>116°02'46" E
**Average**	**395.4**	**-8.47**	**41.18**	**0.335**	**n = 14**
**Stdev**	**7.3**	**0.22**	**0.59**	**0.033**	
Palos Verdes, California, USA (PVD)					
2015/04/11 14:44	409.5	-9.04	40.92	0.333	Latitude:<br>33°42'53" N<br><br>Longitude:<br>118°18'42" W<br><br>Elevation:<br>330 m a.s.l.
2015/04/25 14:24	406.9	-8.88	41.46	0.301	
2015/05/02 13:42	410.6	-8.92	41.46	0.333	
2015/05/09 13:55	407.7	-8.89	41.65	0.290	
2015/05/16 14:33	407.7	-8.96	41.39	0.377	
2015/05/23 14:23	403.3	-8.73	41.70	0.339	
2015/05/30 15:18	405.2	-8.84	41.68	0.325	
2015/06/13 14:11	405.5	-8.87	41.46	0.313	
2015/06/20 14:43	407.6	-8.83	41.37	0.347	
2015/07/04 14:14	400.0	-8.66	41.71	0.295	
2015/07/19 13:56	404.7	-8.67	41.75	0.370	
2015/07/25 14:15	395.4	-8.44	41.32	0.349	
2015/08/08 14:11	391.2	-8.39	41.12	0.277	
2015/08/15 14:07	400.1	-9.52	40.37	0.281	
2015/08/22 14:04	400.3	-8.46	41.20	0.344	
2015/08/29 14:28	405.9	-8.70	41.38	0.330	
2015/09/12 13:49	408.6	-8.80	41.38	0.310	
2015/09/26 14:00	401.1	-8.69	40.83	0.308	
2015/10/10 14:36	405.9	-8.63	41.47	0.312	
2015/10/24 14:45	413.8	-9.33	40.71	0.268	
2015/12/05 14:09	413.6	-10.57	39.32	0.259	
**Average**	**404.8**	**-8.89**	**41.22**	**0.317**	**n = 21**
**Stdev**	**5.7**	**0.47**	**0.56**	**0.032**	

16
